# Combined Toxicities of Di-Butyl Phthalate and Polyethylene Terephthalate to Zebrafish Embryos

**DOI:** 10.3390/toxics11050469

**Published:** 2023-05-19

**Authors:** Qiang Zhang, Wenjie Ma, Jingmin Zhu

**Affiliations:** 1 School of Fishery, Zhejiang Ocean University, Zhoushan 316022, China; 2 Guangxi Key Laboratory of Marine Environmental Change and Disaster in the Beibu Gulf, Beibu Gulf University, Qinzhou 535011, China

**Keywords:** polyethylene terephthalate, di-butyl phthalate, zebrafish embryos, malformation phenotype, transport carrier

## Abstract

The increasing concern for the ecological risks of microplastics (MPs) as carriers of hydrophobic organic contaminants is evident. Di-butyl phthalate (DBP) is extensively utilized as an additive in plastic products, and both DBP and MPs are widespread in the environment. However, the combined toxicity of these substances remains uncertain. In this study, zebrafish embryos were employed to assess the toxic effects of polyethylene terephthalate (PET, MPs) and DBP, with a focus on the DBP toxicities influenced by PET. The embryonic chorion was partially covered by PET particles, and PET led to a delayed hatching of zebrafish embryos without inducing death or teratogenesis. On the other hand, exposure to DBP considerably inhibited the hatching of embryos, leading to severe lethal and teratogenic effects. The most common phenotypes induced by DBP exposure were delayed yolk sac absorption and pericardial edema. The mortality increased in co-treatment with 100 particles/mL PET and 2 mg/L DBP at 24 hpf and 48 hpf. The malformation phenotype, bent notochord, and delayed yolk sac absorption became more severe in 1 mg/L DBP exposition with the co-exposure of 100 particles/mL PET at 72 hpf. PET might act as a carrier that enhances the bioavailability of ambient DBP.

## 1. Introduction

Plastics are widely used in people’s daily lives and are easily discarded. Plastics with diameters between 1 µm and 5 mm are described as microplastics (MPs), which are present in marine systems worldwide and especially concentrated in estuaries, lakes, and coastal waters where humans are abundant [1]. The amount of MPs and plastic debris in the global ocean was estimated to be at least 270,000 tons in 2014 [2], and the most common types were polystyrene (PS), polyethylene terephthalate (PET), and polypropylene (PP) [3]. PET is widely used as synthetic fibers, and the annual global production was approximately 53.3 million tons in 2016 [4,5]. MPs can be ingested by various organisms and are known for vector transport of hydrophobic organic contaminants, and there are growing concerns regarding their potential adverse effects on ecosystems and human health [6,7,8,9,10].

It was reported that MPs could affect photosynthesis and metabolism in phytoplankton [11]. A study by Wu et al. found that MPs could activate the expression of antibiotic-resistance genes and promote the spread of pathogens in microorganisms [12]. MPs can be enriched in the gill, stomach, and gut of organisms, which could be passed along the food chain [13] and cause inflammation [13]. In zebrafish, the toxicities of MPs include developmental delay [14,15], intestinal and metabolism damage [16,17,18], oxidative stress [15,18,19,20], immunotoxicity [15,21], neurotoxicity and locomotor toxicity [15,22,23], genotoxicity [16,22], and reproductive toxicity [22,24]. Studies have also found the effects of microplastics were often more severe during early development. Cormier et al. (2022) found that the exposure of early life stages to particles in water induced a decrease in larval swimming activity [25]. Chronic trophic exposure to MPs reduced growth and reproduction for both fish F0 and survival, growth, and behavior of F1 larval offspring were affected by MPs [25]. Rochman et al. (2014) observed 20 μm PS and PE microplastics present in zebrafish eleutheroembryos (from the larval stage to the onset of active feeding) at concentrations as low as 0.2 mg/L caused growth inhibition and delayed inflating of swim bladders, or their absence [26]. The ingestion of plastic debris at environmentally relevant concentrations may alter endocrine system function in adult fish and warrants further research [26]. Although many studies have reported the toxicity of microplastics in the lab, the actual environmental risks of microplastics and their associated chemicals remain largely unknown [27,28].

Phthalates (PAEs) are used as additives in plastic products to increase elasticity and flexibility and also as carriers of pesticides and insect repellents [29,30,31,32]. PAEs are not chemically bonded to plastic polymers and can easily migrate from products to the environment, having become common in the environment [33,34]. PAEs are not easily degraded and can be enriched in biological tissues [35]. Six PAEs, including dioctyl phthalate (DEHP), diethyl phthalate (DEP), dimethyl phthalate (DMP), benzyl butyl phthalate (BBP), di-n-octyl phthalate (DNOP), and di-butyl phthalate (DBP), have been listed as priority pollutants by the United States Environmental Protection Agency (USERA) [36]. DBP is one of the most commonly used plasticizers and is widely used in children’s toys, plastic food containers, cosmetics, pharmaceuticals, and insect repellents [37]. DBP has been frequently detected in surface waters, wastewater, sewage sludge, sediments, and aquatic organisms worldwide at ng/L to μg/L levels [38,39,40,41,42]. The maximum value of DBP (3.55 μg/L) in a water sample from Yangtze River Delta, China, exceeds the limit value, which implies that there is a potential impact on the environment or human body [43]. The concentrations of DBP in Hangzhou Bay (2.85–18.0 μg/L) and Zhenjiang (0.330–12.6 μg/L) were much higher than those reported in the water in the Jiangsu section in Yangtze River (0.105–0.286 μg/L), the East China Sea (0.088–4.96 μg/L), Taihu Lake (nd–2.54 μg/L), and Yangtze River Delta (nd–7.19 μg/L) [43]. Moreover, the average concentration of phthalate esters (PAEs) in sediment samples was found to be around 1200 μg/kg, which was about 300 times higher than the concentration in surface water samples (4.11 μg/kg). Lee et al. (2019) reported a positive correlation between the concentrations of phthalate esters (PAEs) in sediment and their log Kow values, suggesting that PAEs with higher Kow values have a greater tendency to adsorb to sediment [44]. DBP poses risks to aquatic organisms, even at low levels. DBP can bioaccumulate in the food chain and biomagnify to high levels that threaten fish-eating wildlife and humans. It was reported that exposure to DBP had resulted in yolk sac abnormalities, skeletal defects, spinal curvatures, abnormal movement, craniofacial defects, cardiac defects, defects in eye vascularization, as well as immunotoxicity in zebrafish embryos or larvae [33,45,46,47,48,49].

Due to its hydrophobic properties and large surface area, MPs possess strong adsorption affinity to environmental pollutants, such as POPs and heavy metals [50,51,52]. MPs could increase the neurotoxicity of bisphenol A to zebrafish [53]. A study by Zhang et al. found that the mixture of MPs and Cd resulted in antagonistic toxicity under low concentration of MPs (0.05, 0.1 mg/L), while there was synergistic sublethal toxicity under high levels of MPs (1, 5, 10 mg/L) on zebrafish embryos [3]. Both MPs and DBP are common in the environment, but their combined toxicity is still unclear. Zebrafish is one of the most widely used model species to study the developmental toxicity of chemicals. Zebrafish embryos are more sensitive to environmental pollutants than adult fishes [54,55,56], and the fertilization is external, facilitating toxicant exposure at defined concentrations [57]. The present study aimed to investigate the toxic effects of the combination of polyethylene terephthalate (PET) and di-butyl phthalate (DBP) on zebrafish embryos, with a focus on the potential influence of PET on DBP toxicity.

## 2. Materials and Methods

### 2.1. Chemicals and Reagents

In this study, di-butyl phthalate (DBP) with a purity of 99.7%, tricaine (MS-222) used for anaesthesia, and Dimethyl sulfoxide (DMSO) were procured from Aladdin (Shanghai, China). Colorless polyethylene terephthalate (PET) particles were obtained from the local market and simultaneously sold online at https://m.tb.cn/h.UsDYlDY?tk=ksf3dk4Sw9X (18 May 2023). To aid observation, the PET particles were stained bright red using Nile red (CAS 7385-67-3, Aladdin, Bay City, MI, USA) in the laboratory. Steel sieves with a pore size of 150 μm and 100 μm were used to screen out particles with sizes between 100 μm and 150 μm. Before the exposure experiments, the prepared PET particles were visually inspected and validated through photographs. This approach was taken to ensure that only high-quality particles were used, and to minimize the possibility of any contamination or variability in the results.

### 2.2. Zebrafish Maintenance and Embryo Collection

The zebrafish used in this study were kept in a temperature-controlled room at 28 ± 0.5 ℃ with a 10:14 h dark:light cycle in a closed flow-through system that utilized charcoal-filtered tap water. The experimental procedures followed the OECD guidelines for chemical testing [58]. The fish embryos were collected and examined under a stereo microscope at 4 h post-fertilization (hpf) to ensure their health and viability. We individually weighed 0.294 g of CaCl_2_·2H_2_O, 0.123 g of MgSO_4_·7H_2_O, 0.065 g of NaHCO_3_, and 0.006 g of KCl and dissolved them in 1 liter of fully aerated deionized purified water. After filtration, the solution was used for embryo culture. This ensured that the fish were kept in a healthy and optimal environment throughout the course of the study.

### 2.3. Acute Exposure Experiments of PET and DBP

In the DBP exposure experiment, the low concentration group was set at 0.05 mg/L, which was based on the environmental concentration (~20 μg/L, China) [19]. In the PET exposure experiment, the low concentration group was set at 1 particle/mL. The microplastic pollution in aquaculture water is about 50 particles per liter [59]. Considering the uneven distribution of microplastics in the exposure solution, the exposure concentration was set higher than the environmental concentration. The concentration gradient was set by increasing 1.5–5 times based on the lowest concentration, and a total of 5 exposure concentrations were established. DBP was dissolved in DMSO and then diluted into the embryo culture medium. The medium was filtered through a 0.45 μm filter membrane and maintained at 28 °C for the embryo exposure experiments. Embryos were exposed to 0.1% DMSO and 0.05, 0.2, 1, 2, and 3 mg/L DBP. PET stocks with embryo culture medium were sonicated at 40 kHz for 1 min prior to quantification and use for exposures. The exposure concentration gradient of PET was set at 0, 1, 10, 50, 100, and 200 particles/mL. Exactly 10 mL of suspension was added into a glass Petri dish with 6 cm diameter, and then 20 fertilized zebrafish embryos (screened at 4 hpf) were exposed in each dish and maintained in an incubator at 28 ± 0.5 °C until 96 hpf. Each concentration was tested in quadruplicate, with 20 embryos per replicate, resulting in a total of 80 embryos per concentration. (*n* = 4, 80 individuals). Embryos were observed at 12 hpf, 24 hpf, 48 hpf, 72 hpf, and 96 hpf, and the exposure suspension was changed every 24 h.

### 2.4. Measurement of the Acute Toxicity Indicators

The measurement of the acute toxicity indicators was recorded with reference to Cheng et al. [50]. The acute toxicity indicators included survival, hatching rate, and morphological malformations. Hatching success and survival rates are typically measured at different time points after exposure, and statistical analyses are performed to determine if there are significant differences between exposed and control groups. Morphological abnormalities are evaluated through visual inspection and imaging techniques. In this study, the mortality rate was recorded at 24 hpf, 48 hpf, 72 hpf, and 96 hpf, and the hatching rate was recorded at 48 hpf and 72 hpf. The malformation phenotypes, bent notochord, delayed yolk sac absorption, pericardial edema, and small eyes were analyzed using the image processing software Adobe Photoshop CS4 (CA, USA). After the measurement, the embryos or larvae were returned to the dish for subsequent experiments.

### 2.5. Statistical Analysis

After collecting data from the experiments, statistical analysis was performed using three different software: IBM SPSS 26 Statistics (IBM Corp., Armonk, NY, USA), GraphPad Prism 5.0 (GraphPad Software Inc., San Diego, CA, USA), and Origin 9.0 (OriginLab Corporation, Northampton, MA, USA). The first step in the analysis was to test for the homogeneity of variance, which is the assumption that the variances of the groups being compared are equal. This was done using Levene’s statistic, and the homogeneity value was considered to be greater than 0.05. The mean differences between the control and DBP/PET exposures were then evaluated using one-way analysis of variance (ANOVA) with Dunnett post-hoc test. ANOVA is a widely used statistical method to compare means of three or more groups, and the post-hoc test is used to determine which groups differ significantly from each other. For the comparison of two groups, the independent sample *t*-test was used. This statistical method tests the difference between the means of two independent groups. In the figures, the letters above the bars indicate significant differences, with *p* < 0.05 considered statistically significant. If two groups have the same letter, then they are not significantly different from each other. To ensure the reliability and reproducibility of the results, the experiments were repeated four times. The results were expressed as mean ± SD, which is a standard way of presenting statistical data.

## 3. Results

### 3.1. PET Particles Made for Exposure Experiment

**Figure 1:**
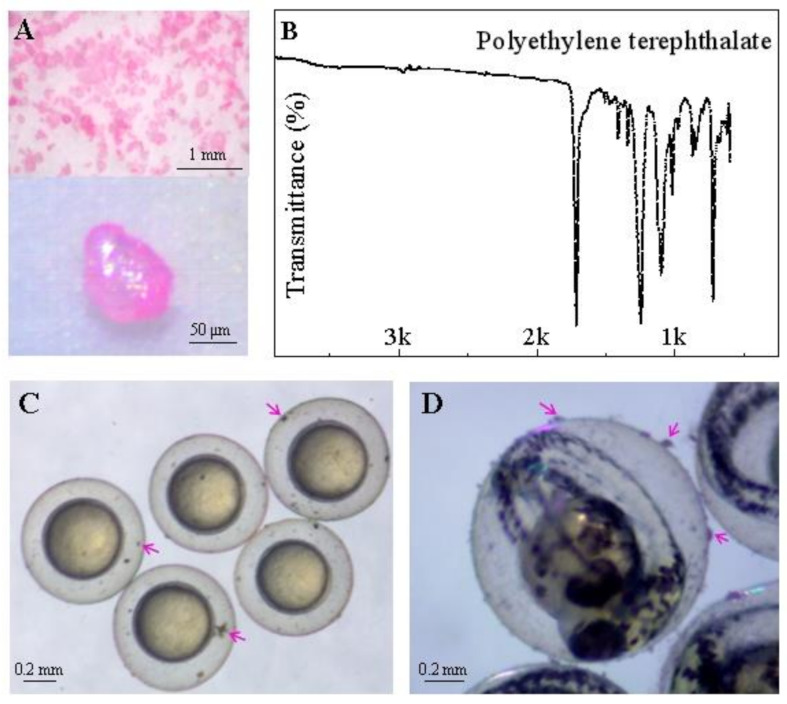
The characteristics of PET particles in this study. (**A**) Morphotype and size distribution of PET; (**B**) Transmittance spectrum analysis of PET; (**C**,**D**) PET particles (arrows) adsorbed on zebrafish embryonic chorion at 6 hpf (**C**) and 48 hpf (**D**).

The prepared PET particles were examined using a Cnoptec SZ680 stereomicroscope (Chongqing, China), and images were captured using an AxioCam digital camera (Figure 1A). The particles were characterized using a micro-Fourier transformed infrared spectroscope (μ-FT-IR, Nicolet iN10 MX, Thermo Fisher Scientific, Waltham, MA, USA) in transmittance mode. The obtained spectrum was compared with the library of polymers provided by Thermo Fisher Scientific in their OMNIC Picta 1 software (Waltham, MA, USA), with a match quality index of >90% (Figure 1B). The images revealed that PET particles could attach to the zebrafish embryonic chorion before the embryo hatched (Figure 1C).

### 3.2. Single Exposure of PET and DBP

**Figure 2:**
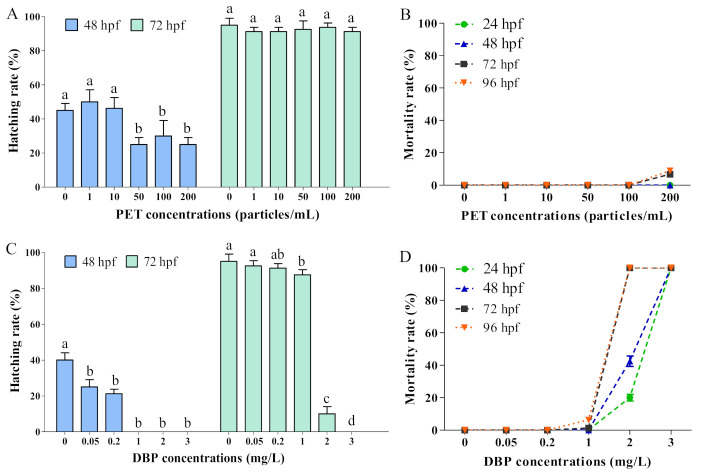
Hatching and mortality rates of zebrafish embryos under PET and DBP expositions. (**A**) Hatching rate at 48 hpf and 72 hpf with PET exposition; (**B**) Mortality rate at 24 hpf, 48 hpf, 72 hpf, and 96 hpf with PET exposition; (**C**) Hatching rate at 48 hpf and 72 hpf with DBP exposition; (**D**) Mortality rate at 24 hpf, 48 hpf, 72 hpf, and 96 hpf with DBP exposition; values represent mean ± SD (*n* = 4); the letters above the bars indicate significant differences (*p* < 0.05). If two arbitrary groups have the same letter, then they are not significantly different.

Zebrafish embryos usually hatch into larvae during 48–96 hpf and mostly hatch at 72 hpf [60]. PET exposure at 50–200 particles/mL significantly delayed the hatching of zebrafish embryos at 48 hpf, while no significant difference was observed at 72 hpf (Figure 2A). A mortality rate of 6–9% was observed in the 200 particles/mL PET group at 72 hpf and 96 hpf (Figure 2B). After being treated with DBP, the hatching rates of embryos with all concentrations were significantly lower than the blank control group at 48 hpf, and inhibition was also observed at 72 hpf with 1–3 mg/L DBP expositions (Figure 2C). DBP exposure at 2–3 mg/L increased the mortality of zebrafish embryos during 24–96 hpf, especially at 72 hpf and 96 hpf, where the mortality reached 100% (Figure 2D).

**Figure 3:**
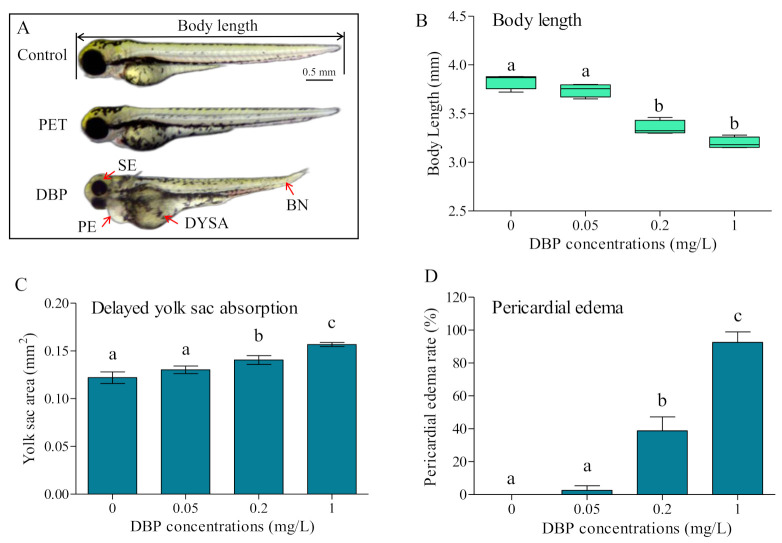
Toxicity effects of PET and DBP singly on the development of zebrafish embryos. (**A**) Embryos under blank control, PET, and DBP expositions; (**B**) Body length of embryos at 72 hpf after DBP exposition; (**C**) Yolk sac area of embryos at 72 hpf after DBP exposition; (**D**) Pericardial edema rates in embryos at 72 hpf after DBP exposition. The letters above the bars indicate significant differences (*p* < 0.05). If two arbitrary groups have the same letter, it signifies that there is no significant difference between them based on the chosen significance level (*p* < 0.05). Abbreviations: BN, bent notochord; DYSA, delayed yolk sac absorption; PE, pericardial edema; SE, small eyes.

No obvious toxic effects on deformity were observed after PET exposure. Exposure to DBP induced severe malformations in zebrafish embryos, including short body length, delayed yolk sac absorption, pericardial edema, bent notochord, and small eyes (Figure 3A). The body length decreased by 2–16% in groups treated with 0.2–1 mg/L DBP compared with the blank control group (Figure 3B). The severity and malformation rate of delayed yolk sac absorption and pericardial edema malformations increased in a DBP concentration-dependent manner (Figure 3C,D).

### 3.3. DBP Toxicities Affected by PET

**Figure 4:**
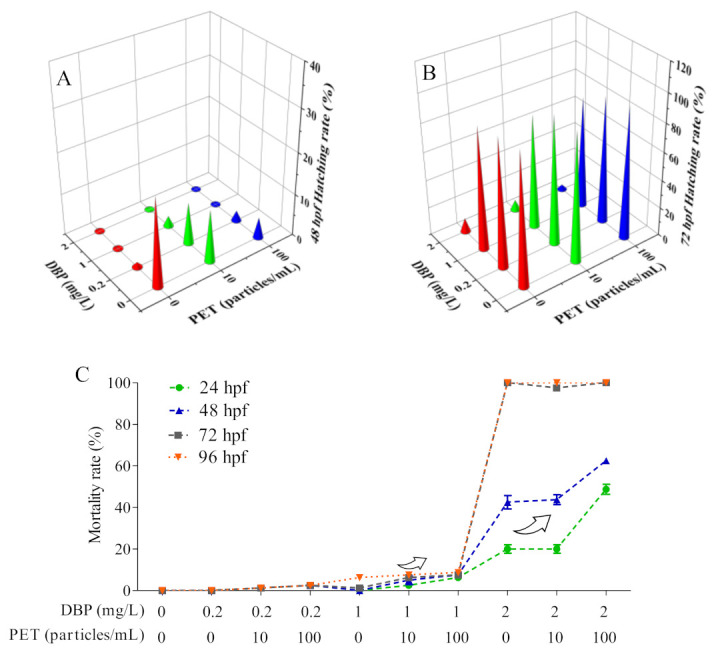
Hatching and mortality rates in zebrafish embryos after co-exposure of DBP + PET. (**A**) Hatching rate at 48 hpf with DBP + PET expositions; (**B**) Hatching rate at 72 hpf with DBP + PET expositions; (**C**) Mortality rate at 24 hpf, 48 hpf, 72 hpf, and 96 hpf with DBP + PET expositions.

A reduction in hatching rate of 10–15% was observed in the 10–100 particles/mL PET groups (Figure 4A). Less than 1% of embryos in the 0.2–2 mg/L DBP groups hatched at 48 hpf, while hatching increased after the addition of PET (Figure 4A). Compared with single DBP exposure, the hatching rate at 72 hpf did not change under DBP + PET exposition (Figure 4B). The mortality increased in co-treatment with 100 particles/mL PET and 2 mg/L DBP at 24 hpf and 48 hpf only (Figure 4C). At 96 hpf, no influence of PET treatment was shown, considering the 100% mortality produced by DBP alone.

**Figure 5:**
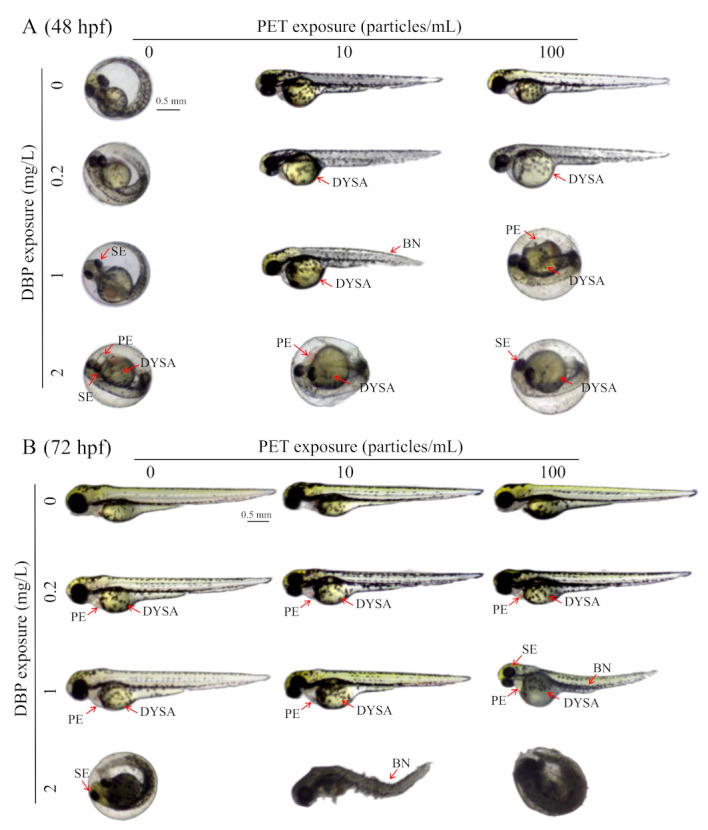
Toxicity effects of DBP + PET co-exposure on the development of zebrafish embryos at 48 hpf (**A**) and 72 hpf (**B**). Abbreviations: BN, bent notochord; DYSA, delayed yolk sac absorption; PE, pericardial edema; SE, small eyes.

After combined exposure to PET, the hatching of zebrafish embryos in 0.2 mg/L DBP treatment was promoted at 48 hpf (Figure 5A). No new malformation phenotype was induced in zebrafish embryos after DBP + PET co-exposure at 48 hpf (Figure 5A). The malformation phenotypes of zebrafish embryos are more diverse and visually apparent at 72 hpf (Figure 5B). The malformation phenotype and delayed yolk sac absorption can still be observed at 72 hpf after DBP exposure. The malformation phenotype, bent notochord, and delayed yolk sac absorption became more severe in 1 mg/L DBP exposition with the co-exposure of 100 particles/mL PET at 72 hpf. The body length of zebrafish embryos in 1 mg/L DBP exposition decreased after co-exposure with 0–100 particles/mL PET at 72 hpf.

## 4. Discussion

In the present study, single exposure to PET did not significantly affect the growth and survival of zebrafish embryos, but delayed hatching at 48 hpf. Zebrafish embryos were surrounded by chorion until the zebrafish larvae hatched out. The chorion of embryos allows small molecules such as water molecules, ions, and oxygen to enter the cells through the membrane pores and prevents large particles of pollutants from entering the cells [61]. Our results showed that the chorionic surface was partly covered by PET particles (Figure 1C). Duan et al. hypothesized that PET particles may affect the permeability of chorionic channels, thereby reducing oxygen delivery, resulting in an anoxic internal microenvironment in zebrafish embryos [62]. PET adsorption can also enhance the mechanical properties of the embryo’s chorionic membrane, which delays embryo hatching [50]. Malafaia et al. found that zebrafish embryos exposed to polyethylene (PE) microplastics induced early hatching compared to controls [14]. The authors speculated this could be due to chorion damage or changes in water quality, such as induction of hypoxia leading to early hatching, but it was not confirmed; however, premature larvae released into the exposed medium did not survive for long. Furthermore, some studies found that larval fish exposed to PE MPs and Polystyrene (PS) nanoplastics also exhibited malformed phenotypes, such as increased yolk sac area, higher head height, pericardium/yolk sac edema, spine curvature, caudal flexure, and larger optic vesicle area [14,63,64,65], but PET exposure did not induce malformed phenotypes in zebrafish larvae, which may be due to different toxic mechanisms of different types of MPs. These adverse effects on early life stages of zebrafish suggest that microplastic pollution poses risks to the development and survival of fish and other aquatic organisms.

Exposure to DBP significantly inhibited embryo hatching (≥1 mg/L) and resulted in severe lethal (≥1 mg/L) and teratogenic effects (≥0.2 mg/L). Sun and Li et al. suggested the inhibition of embryo hatching may be due to DBP inhibiting the secretory function of HCGs, leading to the decrease of hatching enzyme secretion and activity [33]. The prominent phenotypes induced by DBP exposure included delayed yolk sac absorption and pericardial edema. The results of body length showed that DBP exposure had significant inhibitory effects on zebrafish embryo growth. Yolk sac is an important source of nutrition for embryo or larva; physical size will gradually decrease with embryonic development [66]. Delayed yolk sac absorption may result in insufficient nutrient supply, which will inhibit the normal growth of zebrafish. Sun and Li showed that exposure to DBP (1.8, 3.6 μM) led to significantly lower heart rate in zebrafish embryos, which was probably related to the malformations pericardial edema and cardiac structure deformities [33]. It is found that 0.6 mg/L BBP significantly increased the malformation rate, caused growth inhibition, increased the cardiac malformation rate, and reduced the heart rate of embryos [67]. The yolk sac and heart may be the priority targets for DBP toxicity.

Mortality increased in co-treatment with 100 particles/mL PET and 2 mg/L DBP at 24 hpf and 48 hpf. The malformation phenotype, bent notochord, and delayed yolk sac absorption became more severe in 1 mg/L DBP exposition with the co-exposure of 100 particles/mL PET at 72 hpf. As previously mentioned, PET has high hydrophobic properties and can easily accumulate on the surface of embryonic chorion. DBP can adsorb to PET through van der Waals forces and hydrophobic interactions [68,69]. When ingested by marine animals, the DBP adsorbed on PET microplastics may desorb into their tissues, causing endocrine disruption and other adverse effects [26,70]. Cao et al. investigated release behaviors of PAEs from twelve microplastics and found that DBP had the strongest release ability in PA, PP, and PET microplastics (47–84%), and they predicted that about approximately 57.8–16,100 kg/year of PAEs are released into the oceans from microplastics [71]. Thus, DBP might be carried to the embryonic chorion by PET. As PET plastics continue to accumulate in the ocean, they may transport increasing amounts of DBP and exacerbate its ecological impacts. DBP is highly lipophilic and can easily pass through the chorion, resulting in high DBP content in embryos.

The interaction between DBP and PET after being taken up by zebrafish needs to be further investigated. Zhang et al. reported that MPs increased the developmental toxicity of cadmium (Cd) on zebrafish embryos but reduced the lethal toxicity of Cd [3]. The surface characteristics and morphology of PET MPs, such as fibers vs. particles, may influence their affinity for and effects on the embryonic chorionic membrane differently. This could result in different toxic effects on the development of waterborne embryos [50]. The findings from Zhang et al. suggest that interactions between chemicals and MPs are complex, and the toxicity to aquatic organisms depends on multiple factors related to the pollutants and physical properties of MPs. In the present study, the 50–200 particles/mL PET MPs delayed hatching and enhanced 1 mg/L DBP-induced bent notochord and delayed yolk sac absorption phenotypes at 72 hpf, but did not directly cause mortality in zebrafish embryos. PET fibers and particles can differ in specific surface area, which may lead to different levels of adsorption and bioconcentration of DBP. The larger surface area of PET particles could facilitate greater adsorption of DBP compared to fibers, making DBP more available to permeate the chorion upon co-exposure. The toxicity of combined exposure to DBP and PET MPs may thus depend on the type of PET (fiber vs. particle), concentration, and duration of exposure. Further research is required to systematically determine how these parameters influence the joint toxicity of MPs and organic pollutants to aquatic life.

## 5. Conclusions

Our study revealed that PET particles partially covered the embryonic chorion, leading to a decreased hatching rate at 48 hpf. However, PET exposure did not have a significant impact on the growth and survival of zebrafish embryos. In contrast, exposure to DBP caused severe lethal and teratogenic effects, with delayed yolk sac absorption and pericardial edema being the prominent phenotypes. These findings suggest that DBP toxicity primarily targets these two factors. Co-treatment with 100 particles/mL PET and 2 mg/L DBP increased mortality rates at 24 hpf and 48 hpf and resulted in more severe malformation phenotypes such as bent notochords and delayed yolk sac absorption at 72 hpf when exposed to 1 mg/L DBP with the co-exposure of 100 particles/mL PET. It is possible that DBP was carried to the embryonic chorion by PET, which may have increased its bioavailability and, therefore, its toxic effects.

## Data Availability

Not applicable.
